# Prevalence and determinants of discrimination or harassment of women: Analysis of cross-sectional data from Bangladesh

**DOI:** 10.1371/journal.pone.0302059

**Published:** 2024-04-29

**Authors:** M. Iftakhar Alam, Nigar Sultana, Humaira Sultana

**Affiliations:** Institute of Statistical Research and Training, University of Dhaka, Dhaka, Bangladesh; Jatiya Kabi Kazi Nazrul Islam University, BANGLADESH

## Abstract

This study aimed to explore the prevalence and determinants of discrimination against or harassment among Bangladeshi women. The nationally representative cross-sectional data of the Multiple Indicator Cluster Survey 2019 were employed in the study. Women aged 15-49 were asked whether they felt discrimination or harassment due to seven potential reasons in the last twelve months before the survey. The outcome was a binary variable indicating whether a woman has experienced discrimination or harassment for any of the seven reasons. Responses were obtained from 64378 women and analysed through bivariate and multivariate procedures. The data had a hierarchical structure since women were nested within the clusters. A mixed-effects logistic regression model was used to analyse the data appropriately. A strong association between discrimination or harassment was seen with the functional difficulties of women. Notably, women with functional difficulties were 1.629 times more likely to experience discrimination or harassment than women without such difficulties. Other significant factors were the respondents’ area, age, education, current marital status, wealth index quintile and ethnicity. The study concludes that education, marital status, functional difficulties and wealth index are the most important determinants of discrimination or harassment of women.

## Background

Discrimination and harassment are two unavoidable phenomena in today’s world. Discrimination occurs when we differentiate people and give them unequal treatment based on their race, colour, nationality, religion, sex, caste, age, social status, disability, sexual orientation, or genetic information [1]. It has always been a root cause of the ever-widening split in societies. Similarly, harassment involves undesired and offensive conduct of acts which can be ‘physical’, ‘sexual’, or ‘verbal’ [2]. Unfortunately, discrimination or harassment still exists in almost every segment of society. Although people are the victims of discrimination or harassment irrespective of gender, women are the worst sufferers [3].

Various social psychological theories offer explanations for discrimination. These include social cognitive theory, social identity theory, social representations theory, discursive theory, individual differences, conflicts of interest, categorisation processes and identity processes [4]. These theories present diverse viewpoints on the origins of discrimination, encompassing individual-level factors, organisational mechanisms and structural determinants. Discrimination can arise from a range of factors, including age, gender, race, ethnic origin, sexual orientation, physical appearance and social class [5]. The most straightforward method for investigating discrimination involves querying individuals about their encounters with mistreatment due to personal attributes like ethnicity, race, or religion. This can be accomplished by incorporating discrimination-related questions into survey questionnaires. In addition to surveys, alternative approaches to studying discrimination include engaging in ethnographic research or conducting in-depth interviews within potential target groups. Qualitative methods, such as ethnography and in-depth interviews, offer an advantage over surveys as they allow researchers to get more profoundly into the forms, locations and consequences of discrimination [4].

Millions of women worldwide face discrimination and harassment at least once in their lifetime. Mostly, women face discrimination and harassment in the workplace [6]. Discrimination against women occurs mainly in the form of gender discrepancies. In some cases, gender discrimination is followed by sexual harassment because of male chauvinism. Environmental factors such as unbalanced gender ratio and leadership behaviours in job are often responsible for sexual harassment. The prevalence of such harassment is more between two people without a working history than those who have a working relationship of any length [7]. Women who work abroad encounter a higher prevalence of gender harassment compared to their male counterparts. Additionally, there is a positive association between frustration and gender harassment [8]. Hu et al. [9] revealed that instances of discrimination and harassment were prevalent among general surgery residents, particularly affecting women, and were linked to burnout and contemplation of suicide. Some researchers have indicated that male harassers justify their inappropriate conduct by framing it as a religious entitlement. This rationale encompasses penalising women for pursuing professional roles, choosing specific attire, or objectifying women as mere objects meant to satisfy men [10]. According to the study conducted by Rosenbaum et al. [11], 60% of Nepali women encountered verbal, physical, or visual harassment from men in public within a one-year time-frame.

The discrimination and harassment against women hinder their contribution to the economy, leaving them mentally distorted. An investigation in the US evaluated the negative impact of persistent work discrimination and harassment on women firefighter’s physical and mental health, substance abuse and job efficacy, stress and satisfaction [12]. Akhmedshina [13] addressed that discrimination and human rights violation against women is a global problem regardless of social status and cultural level. According to the World Bank, around 2.4 billion working-age women are not afforded an equal economic opportunity, and 178 countries maintain legal barriers that prevent their full economic participation [14].

In Bangladesh, discrimination and harassment is not a new context. However, its magnitude has grown alarmingly in recent years. Women are facing discrimination and harassment in their families, societies and workplaces. Women face glaring discrimination in exercising their legislative powers and are unjustifiably excluded from economically valued assignments of governance [15]. Begum et al. [16] explored the occurrence of sexual harassment among garment workers in Bangladesh. It is found that with low and irregular wages, women workers are also victimised by sexual harassment from their co-workers, goons or police. Several other authors also investigated sexual harassment [17, 18]. In Bangladesh, women’s involvement in politics is increasing remarkably. Despite notably increased female participation in local politics, they are still treated as subordinate to men in many respects [19]. Rahman and Al-Hasan [20] showed that, on average, a woman in Bangladesh earns 12.2% lower wages than a man, and about half of the wage gap can be explained by labour market discrimination against women.

Discrimination and harassment are not inherited in nature. Some socio-cultural factors are responsible for such a heinous act. Zaman [21] dealt with various forms of violence and discrimination and demonstrated their relationships and common roots in Bangladesh. Sultana and Zulkefli [22] examined discrimination against women in two developing countries, Bangladesh and Malaysia. The study was conducted to apprehend the socio-cultural influence of discrimination against women based on their experiences in Bangladesh. Ahmed et al. [23] described an ethnographic study designed to focus groups on sexual harassment with women at three different universities in Dhaka to understand the problem of sexual harassment in the public places of Bangladesh and the associated factors behind it.

The majority of earlier investigations focused on the issue of sexual harassment against women in Bangladesh, without addressing discrimination and harassment based on variables such as ethnicity, age, religion, disability and other factors. More importantly, none of these studies used large-scale national survey data. The sixth round of the Multiple Indicator Cluster Survey (MICS) in Bangladesh collected data on discrimination or harassment against women for the first time. This paper explored these cross-sectional data to learn about the socio-demographic characteristics associated with discrimination or harassment. More specifically, the study aimed to address two key inquiries: Firstly, what is the prevalence of discrimination or harassment among women? Secondly, who is more prone to experiencing discrimination or harassment?

## Materials and methods

### Ethics approval

A technical committee of Bangladesh Bureau of Statistics approved the survey protocol. For all respondents who participated in the survey, verbal consent has been obtained. The voluntary nature of participation, as well as the confidentiality and anonymity of information were communicated to all respondents. In addition, they were informed of the right to refuse any or all questions and that their interview would be terminated at any time. The study utilised secondary data, which was obtained from https://mics.unicef.org/surveys in January 2021. It would be useful to mention that the authors did not have access to information that could identify individual participants during or after data collection.

### Study sample

This paper was based on the cross-sectional data from the sixth round of MICS [24]. The Bangladesh Bureau of Statistics (BBS) surveyed in collaboration with UNICEF Bangladesh in January-May 2019. The nationally representative survey covered various health and well-being issues related to children and women in Bangladesh. The survey included both urban and rural areas of seven administrative divisions. Households in the sample were selected following a two-stage stratification technique. In the first stage, 634 clusters in urban areas and 2586 clusters in rural areas were chosen using a probability proportional to size sampling procedure. In the second stage, a systematic sample of 20 households was selected from each cluster. Although 68711 women aged 15-49 were identified in the survey, 64378 (93.7%) were successfully interviewed. The collected information on these women was available in the women’s dataset, which we utilised in this paper. More on the survey and sampling techniques are available in the final report of MICS 2019 [24].

### Outcome variable

In the MICS, women were asked whether they had felt any form of discrimination or harassment (DH). The specific question asked was, ‘In the past 12 months, have you personally felt discriminated against or harassed on the basis of the following grounds?’ And the grounds were: ethnic or immigration origin, gender, sexual orientation, age, religion or belief, disability, or any other reason. The outcome variable DH chosen for this study was a binary variable with the categories ‘yes’ (for those who encountered any of the seven grounds) and ‘no’ (for those who did not encounter any of the seven grounds).

### Selected covariates

The main covariates of interest were: area (‘rural’, ‘urban’), division (‘Barishal’, ‘Chattogram’, ‘Dhaka’, ‘Khulna’, ‘Mymensingh’, ‘Rajshahi’, ‘Rangpur’, ‘Sylhet’), age (‘15-19’, ‘20-24’, ‘25-29’, ‘30-34’, ‘35-39’, ‘40-44’ and ‘45-49’), education (‘pre-primary or none’, ‘primary’, ‘secondary’, ‘higher secondary+’), currently married (‘yes’, ‘no’), functional difficulties (‘yes’, ‘no’), exposure to mass media (‘yes’, ‘no’), wealth index quintile (‘poorest’, ‘second’, ‘middle’, ‘fourth’, ‘richest’) and ethnicity (‘Bengali’, ‘others’). So all the covariates included for analysis were categorical measures.

The Washington Group on Disability Statistics developed a short set of questions to assess the functioning of women [25]. The questions reflect six domains for measuring functional difficulty: seeing, hearing, walking, self-care, communication and remembering. These questions were included in the individual questionnaire of women in MICS6. The data on these questions were collected directly from the respondents. The ‘functional difficulties’ had been developed as a variable with the categories ‘yes’ (for those who had at least one of the difficulties) and ‘no’ (for those who did not have any of the difficulties).

Exposure to mass media was created based on information from reading a newspaper, listening to the radio, or watching television. If any of these media were used at least once a week by a woman, mass media was ‘yes’. Otherwise, it was ‘no’. The wealth index was a composite measure to indicate a household’s living standard. It was constructed based on selected household assets like owning a television, bicycles, construction materials of household, types of water access and sanitation facilities, etc. This measure was classified into five ordered categories, as indicated earlier. It should be helpful to mention that the index was readily available in the data file.

### Data analysis

The women’s dataset was carefully checked for missing values and outliers. The MICS sample weights were applied in the analyses. The percentage distribution of responses was obtained using univariate statistics. The chi-square tests [26] were performed to examine the association of covariates with the outcome variable. The covariates that showed significant association with the outcome variable were taken to regression analysis.

Because women were sampled from all selected cluster areas in a district, MICS data had the hierarchy. Some common features may appear to be shared between women in a similar cluster and these are likely to lead to responses that correlate each other. In such a situation, it is not possible to capture variability due to clusters by running a fixed-effects logistic regression model. Moreover, it leads to the incorrect standard error of the parameter estimates [27]. For clustered binary responses, we therefore used mixed-effects logistic regression model [28].

*Y*_*ij*_ was the binary response of DH by *j*th woman in *i*th cluster, and *π*_*ij*_ was the probability that a woman experienced DH. Also, *X* = {*X*_1*ij*_, *X*_2*ij*_, …, *X*_*kij*_} denoted the set of *k* observed covariates for *j*th woman in *i*th cluster. Then we fitted the following model to the data

$$\begin{array}{c} \text{log}\;(\frac{\pi_{ij}}{1-\pi_{ij}})=\beta_{0}+\beta_{1}X_{1ij}+\beta_{2}X_{2ij}+\ldots +\beta_{k}X_{kij}+u_{i}, \end{array}$$

where *β*_*k*_ was the coefficient associated with the covariate *X*_*k*_, and *u*_*i*_ was the random effect of *i*th cluster. Note that the considered model was a random intercept model. It was assumed that *u*_*i*_s followed the normal distribution with mean 0 and constant variance $\sigma_{u}^{2}$. The *β*s explained the effect of covariates averaged over the clusters, and random effects *u*_*i*_ adjusted for the cluster-specific mean response. Intra-cluster correlation (ICC) could be obtained using between-cluster and within-cluster variances. The residual variance of women within a cluster was 0 with a constant variance *π*^2^/3 [27]. Therefore, the ICC was defined as

$$\begin{array}{c} \rho =\frac{\sigma_{u}^{2}}{\sigma_{u}^{2}+\frac{\pi^{2}}{3}}. \end{array}$$

Since *ρ* is the ratio of between-cluster and within-cluster variations, *ρ* close to zero means no variation in the responses due to the clusters. In contrast, *ρ* > 0 means there is a variation in responses due to the clusters. That is, *ρ* > 0 indicates a correlation among the binary responses within the clusters. Results of the regression analysis were presented in terms of odds ratio (OR) with a corresponding 95% confidence interval (CI). The data management and analysis were done using Stata version 14 [29].

## Results

The percentage distribution of women by their background characteristics is shown in Table 1. The column prior to the last two shows percentages for DH, that is, those who felt discriminated against or harassed for any of the seven reasons. The majority of the women in the sample were from rural areas. Age appeared to be the most common reason for discrimination or harassment (3.8%), followed by gender (3.7%) and sexual orientation (3%). Of the women, 10.5% experienced DH for any of the listed seven reasons. While 9% women experienced DH in the urban areas, it was 10.9% in the rural areas. The prevalence of women varied widely among the divisions, from 6% in Sylhet to 20.4% in Mymensingh. There were also considerable variations across the age groups, with the highest in the age group 15-19 (14%) and the lowest in group 45-49 (8%). More importantly, the percentage decreases as we move to a higher age group. The prevalence across the levels of education did not vary substantially. While 8.9% of currently married women experienced DH, it was 16.6% among the women who were not married then. There were also considerable variations in the percentage at the levels of functional difficulties. The percentage of DH was higher among the women with functional difficulties (15.5%) than those with no such difficulties (9.8%).

**Table 1:** Percentage of women aged 15-49 years who in the last 12 months felt discriminated against or harassed, Bangladesh, 2019.

Characteristic	Ethnic or immigration origin	Gender	Sexual orientation	Age	Religion	Disability	Other reason	DH	*P*-value	Number of women
Total	1.7	3.7	3	3.8	1.5	1.2	0.5	10.5		64378
Area									< 0.001	
Urban	1.5	2.7	2.8	3.3	1.5	0.9	0.3	9		15094
Rural	1.7	4	3	4	1.5	1.3	0.6	10.9		49284
Division									< 0.001	
Barishal	0.3	1.4	3.1	6.4	1.3	3	0.9	12.2		3465
Chattogram	1.6	1.7	4	3.6	1.1	0.9	0.3	8.5		12514
Dhaka	1.5	2	1.7	3.1	1.7	0.8	0.3	7.9		16316
Khulna	0.8	4.6	4.1	3.3	0.9	1.1	0.4	10.4		7578
Mymensingh	1.7	13.1	4.7	7.3	2	1.2	1	20.4		4181
Rajshahi	2.4	7.6	3.5	4.1	1.7	1.6	1.2	15.6		8521
Rangpur	2.6	2.8	2.3	3.2	2.2	1.4	0.3	9.8		7081
Sylhet	1.7	1.4	0.8	3.2	0.6	1	0.2	6		4722
Age									< 0.001	
15-19	1.4	4.4	6.6	5.1	1.5	1	0.4	14		11950
20-24	1.5	4	3.1	4.5	1.6	1.1	0.5	11.3		10404
25-29	1.8	3.6	2.5	4.2	1.3	1.3	0.5	10.6		10031
30-34	1.7	3.7	2	3	1.2	1.3	0.7	9.3		10224
35-39	1.8	3.4	1.7	3.2	1.7	1.3	0.5	9.2		9206
40-44	1.7	3.5	1.5	2.9	1.4	1.1	0.6	8.3		6788
45-49	1.9	2.9	1.1	3	1.5	1.3	0.4	8		5776
Education									0.300	
Pre-primary or none	2.3	4.1	1.8	3.9	1.6	1.7	0.6	10		10187
Primary	2	4	2	3.9	1.5	1.7	0.7	10.8		14615
Secondary	1.5	3.6	3.5	3.7	1.4	0.9	0.5	10.5		28497
Higher secondary+	0.8	3.2	4	4.1	1.4	0.8	0.4	10.3		11079
Currently married?									< 0.001	
Yes	1.7	3.3	1.9	2.9	1.4	1.1	0.5	8.9		51121
No	1.6	5.5	7.1	7.5	1.7	1.6	0.6	16.6		13256
Functional difficulties									< 0.001	
Yes	1.6	4.5	2	5	1.6	7.1	1.2	15.5		1760
No	1.7	3.6	2.4	3.6	1.5	1	0.5	9.8		55886
Exposure to mass media									< 0.001	
Yes	1.4	3.5	3.1	3.5	1.5	1	0.5	10.1		41821
No	2.1	4.1	2.7	4.4	1.5	1.5	0.6	11.2		22557
Wealth index quintile									< 0.001	
Poorest	2.5	4.7	2.9	5	1.7	2.1	1	13.1		11267
Second	2.1	4.9	2.9	4.4	1.7	1.3	0.7	12.3		12327
Middle	1.4	4	3.3	4.1	1.4	1.1	0.5	10.8		12988
Fourth	1.5	3.3	3.1	3.4	1.6	1	0.2	9.8		13625
Richest	1	2.1	2.6	2.5	1.1	0.6	0.2	7.1		14170
Ethnicity									0.762	
Bengali	1.6	3.7	3	3.8	1.4	1.2	0.5	10.5		63626
Others	4.4	4	1.7	3.3	3.5	1.1	0.5	10.1		752

Of the women who had media exposure, 10.1% of them experienced DH. There was a little increase in percentage (11.2%) among the women without such exposure. A wide disparity was observed among the women in wealth index quintiles: 13.1% women in the poorest quintile compared with only 7.1% from the richest quintile. Most of the respondents in the study sample were of Bengali origin. However, the percentage of Bengali women who experienced DH was not appreciably different from that of other origins. The column before the last one shows the *P*-values obtained from the chi-square test of each covariate with the outcome variable DH. Each covariate, except ‘education’ and ‘ethnicity’, was highly associated with the outcome variable, as reflected through the small *P*-values.

Table 2 shows the results obtained for the mixed-effects logistic regression model to assess the determinants of DH. Since education and ethnicity were found not to be associated with the outcome variable, initially, we thought to refrain them from the regression model. However, they showed a significant association with the outcome variable during the regression analysis, and therefore, we kept them in the model. Area of residence was significantly associated with the outcome variable DH. Women in urban areas were 10% more likely (OR 1.100; 95% CI 1.011-1.196) compared with their rural counterparts to experience any form of discrimination or harassment. The experience of DH was not statistically significant across the divisions. Older women were significantly less likely to experience DH. The likelihood of DH was high in the age group 20-34 compared to those at 15-19. However, the likelihood decreased in the subsequent age groups. Women aged 45-49 were 31.8% less likely to have DH than those aged 15-19.

**Table 2:** Factors associated with discrimination or harassment women of age 15-49 years in Bangladesh, 2019.

Characteristic	OR	*P*-value	95% CI for OR
Area (Ref: Rural)			
Urban	1.100	0.025	1.011, 1.196
Division (Ref: Barishal)			
Chattogram	0.819	0.673	0.326, 2.061
Dhaka	0.967	0.942	0.394, 2.369
Khulna	0.970	0.950	0.380, 2.475
Mymensingh	1.747	0.350	0.543, 5.592
Rajshahi	2.109	0.134	0.795, 5.596
Rangpur	1.135	0.799	0.427, 3.018
Sylhet	0.455	0.189	0.140, 1.472
Age (Ref: 15-19)			
20-24	1.113	0.059	0.995, 1.245
25-29	1.132	0.037	1.007, 1.274
30-34	1.046	0.459	0.928, 1.181
35-39	0.959	0.522	0.846, 1.088
40-44	0.780	<0.001	0.679, 0.896
45-49	0.682	<0.001	0.589, 0.791
Education (Ref: Pre-primary or none)			
Primary	1.028	0.564	0.936, 1.128
Secondary	0.863	0.003	0.783, 0.950
Higher secondary+	0.779	<0.001	0.689, 0.882
Currently married? (Ref: No)			
Yes	0.350	<0.001	0.323, 0.380
Functional difficulties (Ref: No)			
Yes	1.629	<0.001	1.415, 1.874
Exposure to mass media (Ref: No)			
Yes	1.005	0.858	0.939, 1.077
Wealth index quintile (Ref: Poorest)			
Second	0.867	0.002	0.794, 0.947
Middle	0.798	<0.001	0.726, 0.879
Fourth	0.772	<0.001	0.695, 0.857
Richest	0.604	<0.001	0.529, 0.687
Ethnicity (Ref: Others)			
Bengali	0.718	0.048	0.517, 0.997
Constant	0.329	0.008	0.145, 0.747

Secondary education was associated with women’s experience of DH. Women who had attended secondary school were 13.7% less likely to experience DH than those with pre-primary or no education. This likelihood further decreased when women had higher secondary or more education. Currently married women were 65% less likely to face DH than those who were not married (OR 0.350; 95% CI 0.323-0.380). Functional difficulties of women was significantly associated with experiencing DH. Compared with women with no functional difficulty, women with functional difficulties were 1.629 times more likely to be exposed to DH (OR 1.629; 95% CI 1.415-1.874). Exposure to mass media was not significantly associated with DH. The likelihood of women getting discriminated against or harassed decreased monotonically with the wealth index. The richest women were 39.6% less likely than the poorest women to experience DH (OR 0.604; 95% CI 0.529-0.687). Regarding ethnicity, Bengali women were 28.2% less likely than those of other origins (OR 0.718; 95% CI 0.517-0.997). The estimate of the variance of random effects (${\hat{\sigma }}_{u}^{2}$) was obtained as 0.82. The resulting intra-cluster correlation was $\hat{\rho }=0.2003$. That is to say, 20.03% of the variation in the outcome variable DH were due to the variation in clusters.

## Discussion

The experience of DH among women was found to be more prevalent in urban areas than in rural areas, which is consistent with Ahmed and Maitra [30]. One possible explanation for this phenomenon could be that women residing in urban areas tend to possess a higher level of awareness. As a result, instances of discrimination or harassment against women may be more widely recognised and reported. Age had a significant association with the outcome variable. Younger women were more discriminated against and harassed. It might be because they were often less conscious of their surroundings and tended to be quieter. The probable reason for elderly women to be less discriminated against may be the maturity of comprehending and coping with situations that come with age.

Compared to pre-primary or none, having primary education did not make a significant difference. However, the likelihood of DH decreased with a higher level of education. Education has the potential to empower women by providing them with knowledge, skills and critical thinking abilities. When women are educated, they are better equipped to challenge DH. Women, who were married, had less likelihood of being discriminated against or harassed. While marriage itself does not inherently shield women from discrimination, certain circumstances may contribute to fewer instances of discrimination. Married women may have social support systems within their families, and this support can help mitigate certain forms of discrimination or harassment.

The strongest association was found between DH and functional difficulties of women. Physically or mentally challenged women endured more discrimination or harassment than others. This finding is consistent with Baldwin and Johnson [31], who estimated the effect of disabilities on the wage of women. Women with functional difficulties may face discrimination due to negative attitudes and stereotypes about disability. Of note, no significant association was found between DH and women’s exposure to mass media once the other covariates had been controlled.

The wealth index had a profound association with DH: the higher the level of the wealth index quintile was, the lower the likelihood of DH among women. Although wealth may offer specific benefits and opportunities, it does not ensure complete protection from discrimination or harassment, and various forms of discrimination or harassment can still endure within different socioeconomic levels. Financial resources contribute to women’s economic autonomy to some extent. Affluent women typically have entry to influential social networks, allowing them to wield social influence and consequently contest discriminatory practices in various domains.

## Strengths and limitations

The study’s main strength was that it was based on large-scale, nationally representative survey data. However, it also had some limitations. Since the study was based on cross-sectional data, it was able to suggest associations rather than causal relationships. Women reported information about DH, and verifying the responses from any other source was impossible. Since they reported events that occurred in the last twelve months, the responses were subject to recall and reporting bias. As typical with most surveys, the questions may not have been understood the same way by all women. Although several potential covariates were included in the study, the survey did not collect information on some other variables. For instance, the employment status of women, place of experiencing DH, etc. Since the study only focused on women, direct comparisons with men in the same settings were not possible.

## Conclusions

This paper revealed that almost eleven percent of women encountered discrimination or harassment in the past twelve months of the survey. The results indicated that factors such as age, education, marital status, functional difficulties, wealth index, and ethnicity were significantly associated the occurrence of discrimination or harassment. The study emphasises the potential of higher education in diminishing the likelihood of DH and highlights the importance of altering societal perceptions towards women facing functional challenges. Additionally, improving the socio-economic conditions of households is crucial for reducing the likelihood of discrimination or harassment. Reducing discrimination or harassment against women necessitates a comprehensive approach that encompasses both individual and collective actions, spanning societal and institutional levels. By tackling underlying issues, we can strive towards fostering a fairer and more inclusive world.
